# Efficient Production of Naringin Acetate with Different Acyl Donors via Enzymatic Transesterification by Lipases

**DOI:** 10.3390/ijerph19052972

**Published:** 2022-03-03

**Authors:** Yesol Baek, Seungmee Lee, Jemin Son, Taek Lee, Jong-Min Oh, Sang Hun Lee, Hyun Uk Kim, Sang Woo Seo, Si Jae Park, Hah Young Yoo, Chulhwan Park

**Affiliations:** 1 Department of Chemical Engineering, Kwangwoon University, Seoul 01897, Korea; isss9511@naver.com (Y.B.); 98lsm@naver.com (S.L.); wkqh14@gmail.com (J.S.); tlee@kw.ac.kr (T.L.); 2 Department of Electronic Materials Engineering, Kwangwoon University, Seoul 01897, Korea; jmoh@kw.ac.kr; 3 Department of Chemical and Biological Engineering, Hanbat National University, Daejeon 34158, Korea; sanghunlee@hanbat.ac.kr; 4 Department of Chemical and Biomolecular Engineering, Korea Advanced Institute of Science and Technology (KAIST), Daejeon 34141, Korea; ehukim@kaist.ac.kr; 5 School of Chemical and Biological Engineering, Institute of Chemical Process, Seoul National University, Seoul 08826, Korea; swseo@snu.ac.kr; 6 Division of Chemical Engineering and Materials Science, Ewha Womans University, Seoul 03760, Korea; 7 Department of Biotechnology, Sangmyung University, Seoul 03016, Korea

**Keywords:** antioxidant, flavonoid, naringin, flavonoid ester, flavonoid acetate, acylation, lipase, transesterification, enzymatic synthesis

## Abstract

Naringin, one of the citrus flavonoids and known as a natural antioxidant, has limited bioavailability owing to its low stability and solubility. However, naringin esters formed via acylation have recently been reported to possess improved physical and chemical properties. The development of these compounds has a great potential in the food, cosmetic and pharmaceutical industries, but low conversion and productivity are barriers to industrial applications. This study aimed to improve the conversion of naringin acetate, which is formed via the enzymatic reaction between naringin and an acyl donor. An optimal reaction condition was determined by evaluating the effect of various variables (enzyme type, enzyme concentration, acyl donor, molar ratio of reactants, reaction temperature, and solvent) on the synthesis of naringin acetate. The optimal condition was as follows: 3 g/L of Lipozyme TL IM, molar ratio of 1:5 (naringin:acyl donor), reaction temperature of 40 °C, and acetonitrile as the reaction solvent. Under this condition, the maximum conversion to naringin acetate from acetic anhydride and vinyl acetate was achieved at approximately 98.5% (8 h) and 97.5% (24 h), respectively. Compared to the previously reported values, a high conversion was achieved within a short time, confirming the commercial potential of the process.

## 1. Introduction

Flavonoids are naturally occurring antioxidants with more than 8000 types identified to date. Based on their structure, they are classified as flavones, flavonols, flavanones, flavanonols, isoflavones, and anthocyanins. Flavonoids are generally found in fruits, vegetables, flowers, and tea [1], and are widely regarded as preventative and therapeutic agents for chronic diseases owing to their biological and pharmacological activities [2,3]. Naringin is a glycosylated flavonoid with rhamnose and glucose attached to the C-7 position of naringenin, an aglycone form. Naringin, a typical citrus flavonoid widely found in grapefruits and citrus peels [4], exerts pharmacological effects, including strong antioxidant [5], antibacterial [6], and anticancer [7] properties, as well as blood and cholesterol-lowering effects [8]. Naringin has advantages over aglycone flavonoids, which are limited in application owing to their mutagenic and cytotoxic properties [9]. Indeed, several studies have reported naringin supplementation as a suitable treatment for obesity, diabetes, hypertension, and metabolic syndrome [4]. Meanwhile, the *Citrus* genus is one of the most important genera worldwide, with a global distribution of up to 90 million tons per year resulting in a significant production of citrus peel waste [10]. However, due to their high flavonoid content, increasing attention has been directed to improving their application in various industries.

Glycosylated flavonoids are generally limited in their bioavailability, owing to their low solubility and stability in hydrophobic environments. These limitations hinder their application from in vitro to in vivo systems [11]. The antioxidant effect and radical scavenging ability of flavonoids depend on their chemical structure as well as their lipophilicity [12]. In addition, glycosylated flavonoids are unstable due to the presence of many hydroxyl groups and are, thus, readily decomposed by light, oxygen, and high temperatures [2]. It is therefore necessary to improve the solubility of naringin in the lipid phase to increase its absorption and stability in physiological conditions. Acylation may be a strategy to increase the solubility of naringin. As chemical acylation is performed at high temperature or high pressure, it is difficult to esterify unstable substances, such as polyols, by this method. In addition, protection and deprotection steps are required to perform the regioselective acylation of flavonoids like polyol [13]. Considering that these processes often generate toxic by-products and require harsh reaction conditions, including high temperatures [14], the application of chemical methods in food has remained limited owing to the adverse effects on the environment and overall safety concerns. In contrast, enzymatic methods use mild conditions and one-step synthesis reactions, without the need for protection and deprotection steps. Moreover, it is an eco-friendly synthesis method, as it generates less by-products due to its excellent regioselectivity [14,15,16].

Esters, such as triacylglyceride (TAG), phospholipid derivatives of phenolic acids, and flavonoid esters, can be produced by enzymatic methods [17,18]. The enzymatic acylation of flavonoids has been studied using proteases, esterases, acyltransferases, and lipases [14,19]. Currently, lipases are used in most flavonoid ester synthesis studies. Lipase is a well-known enzyme with potential in biological processes owing to its availability and stability in both organic and aqueous phases. In particular, microbial lipase is stable in organic solvents, does not require cofactors, and has broad specificity. Therefore, it has been applied to various industries based on the findings of many studies on its reaction mechanism and structure [15,20,21,22].

There are several drawbacks associated with enzymatic synthesis. Besides being expensive, enzymes are proteins and, therefore, prone to denaturation in response to environmental conditions; moreover, their separation and purification are difficult [20,21,22,23]. Immobilization may overcome these shortcomings of free enzymes. When an immobilized enzyme is used, it can be readily separated and reused, thus reducing the overall consumption of the enzyme. Further, the stability against heat and organic solvents is increased [14,15,24], as a result of which many recent studies on the synthesis of flavonoid esters have been conducted using immobilized lipases [20,21]. Existing studies on flavonoid ester, including those on naringin acetate, focus on synthesis using enzymatic methods (Table 1). Synthesis via esterification requires a long reaction time, and the conversion is relatively low. Meanwhile, in transesterification synthesis, the reaction is conducted using either a high molar ratio of the reactants or a large amount of enzyme. A previous study reported the synthesis and statistical optimization of flavonoid acylation using esculin and linseed oil [25], the results of which showed that a conversion of 78.5% could be obtained after 96 h of reaction time. In the case of naringin acetate, a study reported a conversion of 41.03% after 96 h of reaction between naringin and vinyl acetate [10].

Many reports have suggested that acylated flavonoids have improved physical and chemical properties, such as thermal stability, light resistance, and lipophilic solubility [14,30,31,32,33,34,35]. The improved function of acylated flavonoids increases its applicability in various industries, including food, pharmaceutical, and cosmetics. Foods containing flavonoids have a bitter and astringent taste; however, acylated flavonoids have a more favorable taste, that can be applied to food or cosmetics such as toothpaste [30]. In the pharmaceutical industry, acylated flavonoids have been found to be effective in preventing or treating diseases related to hyperglycemia, such as hyperlipidemia and stroke [31]. Additionally, flavonoids acylated with polyunsaturated fatty acids reduce the vascular endothelial growth factor (VEGF) of K562 human leukemia cells and, thus, may represent effective anti-tumor agents [32]. Moreover, Hattori et al. synthesized naringin ester using Lipozyme RM IM and investigated its anti-inflammatory effects [33]. They found that naringin ester exhibited superior anti-inflammatory properties compared to naringin. In addition, flavonoid esters exerted better skin protection properties, protecting against UV-radiation-induced mitochondrial or nuclear DNA damage, compared to flavonoids. As such, they have been reported to protect the skin and scalp from aging and inflammation [34]. Among the various acyl donors, short chains such as an acetyl group were found to improve transport through the aqueous environment, along with its interaction or penetration through phospholipid membranes [35].

Li et al. synthesized naringin ester, including naringin acetate, using whole cells and confirmed its antioxidant effect [10]. Their results confirmed that naringin acetate has a higher free radical scavenging capacity for 2,2-diphenyl-1-picrylhydrazyl (DPPH) and 2,2′-azino-bis (3-ethylbenzothiazoline-6-sulfonic acid) (ABTS) than naringin. In addition, naringin acetate was found to be more effective than vitamin E, as per an oxygen radical absorbance capacity (ORAC) analysis.

To the best of our knowledge, despite many studies on the synthesis of naringin acetate, few have examined the enzymatic synthesis of naringin acetate for high conversion through the optimization approach. Therefore, to improve the conversion and reaction time in the enzymatic synthesis of naringin acetate, we aimed to identify the optimal synthesis conditions by investigating the effects of different types of lipases, concentrations of lipases, types of acyl donors, molar ratio of reactants, reaction temperature, and solvents.

## 2. Materials and Methods

### 2.1. Materials

Naringin was purchased from Sigma-Aldrich (St. Louis, MO, USA). Acetic acid, acetic anhydride, methyl acetate, vinyl acetate, propyl acetate, butyl acetate, acetone, *tert*-butyl alcohol, *tert*-amyl alcohol, and 1,2-dichloroethane were purchased from Dae-Jung (Gyeonggi-do, Korea). Ethyl acetate, acetonitrile, and tetrahydrofuran (THF) were purchased from Junsei (Tokyo, Japan). For the immobilized enzyme, commercially used Novozym 435 (*Candida antarctica* lipase B immobilized on acrylic resin), Lipozyme TL IM (*Thermomyces lanuginosus* immobilized on a silica gel carrier), and Lipozyme RM IM (*Rhizomucor miehei* immobilized on a resin carrier) were selected, purchased from Novozymes (Bagsværd, Demark).

### 2.2. Enzymatic Synthesis of Naringin Acetate

**Figure 1:**
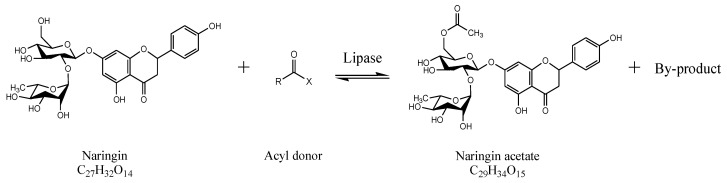
Reaction formula for the production of naringin acetate from naringin and acyl donors.

Naringin acetate was synthesized through an enzymatic reaction (Figure 1). Before the reaction, the organic solvent was dried for more than one week using 3-Å molecular sieves (150 g/L). Naringin was dried for over one week in desiccators with silica gel. Naringin (10 mM) and the acyl donor (10–110 mM), dissolved in an organic solvent, were added to a 50 mL serum bottle in a 20 mL working volume. A certain amount of enzyme (1–9 g/L) was then added. The serum bottle was sealed to prevent the evaporation of the organic solvent, and the mixture was allowed to react in a shaking incubator at 180 rpm for 48 h.

### 2.3. Optimization of Reaction Conditions

**Figure 2:**
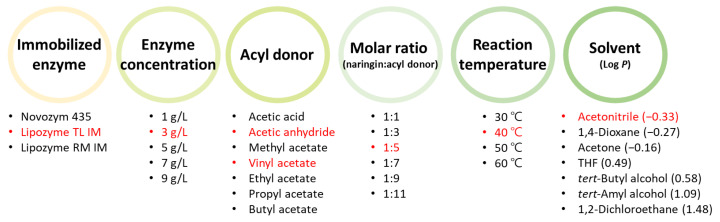
Optimization procedure to produce naringin acetate using naringin and acyl donors. (Red highlighted points: determined variables for the synthesis of naringin acetate).

Optimization was performed using the one factor at a time (OFAT) method, in which only one variable is manipulated at a time, keeping the rest fixed, and optimizing step by step. The effects of six reaction parameters (type of immobilized enzyme, concentration of enzyme, type of acyl donor, molar ratio of reactants, reaction temperature, and solvent) on the synthesis of naringin acetate were investigated (Figure 2). As the basic reaction condition, 5 g/L of the enzyme, vinyl acetate as the acyl donor, 1:1 molar ratio of reactants, reaction temperature of 40 °C, and *tert*-amyl alcohol as the solvent were used. The reaction was conducted in a shaking incubator at 180 rpm for 48 h. Starting with these basic conditions, the factors selected in each stage were applied to the next stage.

To confirm the effect of the immobilized enzyme type on conversion, three types of immobilized lipases (Novozym 435, Lipozyme TL IM, and Lipozyme RM IM) were screened. At this time, the conditions were basic reaction conditions. Thereafter, the enzyme concentration (Lipozyme TL IM) was varied between 1 and 9 g/L. In acyl donor selection, 3 g/L of Lipozyme TL IM was used to screen for acetic acid, acetic anhydride, methyl acetate, vinyl acetate, ethyl acetate, propyl acetate, and butyl acetate. The molar ratio of the reactants was investigated in the range of 1:1 to 1:11 with vinyl acetate or acetic anhydride as the acyl donor. The reaction’s temperature was investigated over the range 30–60 °C, and the molar ratio of the reactants was 1:5. Finally, various organic solvents (acetonitrile, 1,4-dioxane, acetone, THF, *tert*-butyl alcohol, *tert*-amyl alcohol, and 1,2-dichloroethane) were evaluated.

### 2.4. Analytical Methods

High performance liquid chromatography (HPLC) was used for the quantitative analysis, and performed using Agilent 1260 infinity II (Agilent, CA, USA). After completion of the reaction, a sample was collected using a 1 mL syringe. The collected solution was 10-fold diluted with methanol, and the enzyme and residue were removed using a syringe filter, and finally injected into a 2 mL vial. The column used for this analysis was the INNO Column C18 (120 Å, 5 μm, 4.6 × 250 mm). The injection volume was 5 μL, and the column temperature was maintained at 50 °C. As mobile phases, 3% acetic acid in water (A) and 100% methanol (B) were used. The flow rate was 1 mL/min, and the gradient was as follows: (A/B) 0 min-70/30, 5 min-0/100, 10 min-0/100, 15 min-70/30, and 20 min-70/30. The analysis was performed at 280 nm using an UV detector. The conversion was calculated from the initial naringin concentration and the synthesized naringin acetate concentration, as demonstrated in Equation (1) [12,36,37,38]. The naringin calibration curve was obtained using methanol. Error bars and error ranges represented the standard deviations (*n* = 2).

(1) $$Conversion\;\left(\%\right)=\frac{Naringin\;acetate\;concentration}{Initial\;naringin\;concentration}\times 100\;\left(\%\right)$$

Liquid chromatography-mass spectrometry (LC-MS) was used for the qualitative analysis, and was performed using Agilent 1260 Infinity II and Infinity Lab LC/MSD (Agilent, CA, USA). The solution collected for analysis was 100-fold diluted with methanol, filtered, and injected into a 2 mL vial. The injection volume, column, and column temperature were the same as in the HPLC analysis method. The mobile phase contained 0.1% formic acid in water (A) and 0.1% formic acid in acetonitrile (B). The flow rate was 1 mL/min, and the gradient was as follows: (A/B) 0 min-70/30, 5 min-0/100, 10 min-0/100, 15 min-70/30, and 20 min-70/30. The presence of naringin was confirmed by the SIM mode (*m*/*z* 603.20), and that of naringin acetate was confirmed by the SCAN mode. The mass spectrum was scanned in the 600–700 *m*/*z* range.

## 3. Results and Discussion

### 3.1. Synthesis of Naringin Acetate by Transesterification with Acyl Donors

**Figure 3:**
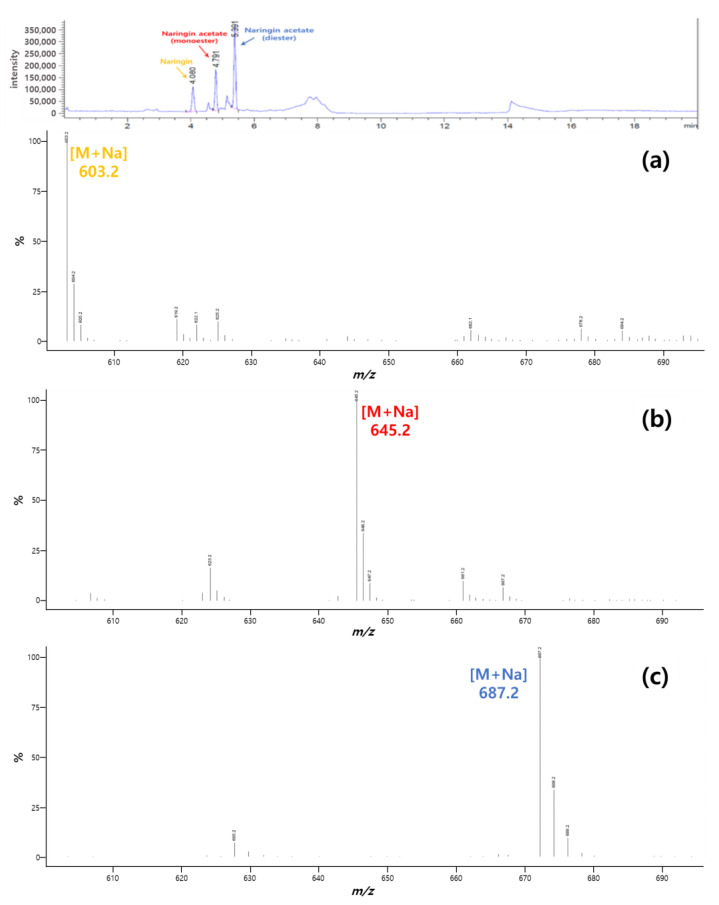
Results of the LC-MS analysis of naringin acetate: (**a**) naringin; (**b**) naringin acetate (monoester); (**c**) naringin acetate (diester).

In this study, naringin acetate was synthesized by the acylation of naringin in an organic solvent. A quantitative analysis of the synthesized naringin acetate was performed by HPLC. Most of the studies to date have reported naringin acylation to occur in the 6″-OH portion of glucose, the primary alcohol [39,40,41,42], resulting in the production of only monoester [43]. As a result of this study, when vinyl acetate was used similar findings were observed as that of the previous study results. However, when acetic anhydride was used, different results were obtained. LC-MS was performed for qualitative analysis; its results showed [M + Na] in all cases (Figure 3). Diester was formed at *m/z* 687.2 and was seen as a new peak when acetic anhydride was used. A similar diester was produced in a previous study [12]. It was reported that acylation may occur in the 6″-OH and 4‴-OH portions of the rhamnoglucoside of naringin.

### 3.2. Selection of Enzyme

Novozym 435, Lipozyme TL IM, and Lipozyme RM IM were screened to identify the enzyme best suited for naringin acetate synthesis. Lipases are generally classified according to their origin or specificity, with the latter including substrate specificity, regioselectivity, and stereospecificity. Novozym 435 originated from *Candida antarctica* lipase B (CALB), which is nonspecific, and was immobilized on a hydrophobic carrier acrylic resin. Lipozyme TL IM is a 1,3-specific lipase from *Thermomyces lanuginosus*, immobilized on silica gel. Lipozyme RM IM is a 1,3-specific lipase from *Rhizomucor miehei*, immobilized with a weak anion-exchange resin based on a phenol-formaldehyde copolymer.

When Novozym 435, Lipozyme TL IM, and Lipozyme RM IM were used, conversions of 17.06%, 29.89%, and 19.91% were obtained, respectively (Table 2). Lipozyme TL IM showed the highest conversion, which is consistent with other studies wherein naringin ester or flavonoid ester showed a high conversion with a Lipozyme TL IM catalyst [12,14,32,44,45]. Lipozyme TL IM is very effective in transesterification and is known to have high substrate selectivity for bulky groups in alcohol and acid moieties [46]. Meanwhile, Novozym 435 shows a better stability in the presence of low-molecular alcohol [47]. However, Lipozyme TL IM is eight and ten times less expensive than Lipozyme RM IM and Novozym 435, respectively [14,48]. Therefore, Lipozyme TL IM was considered a suitable enzyme from an efficient and economical viewpoint.

### 3.3. Effect of Enzyme Concentration on the Conversion of Naringin Acetate

**Figure 4:**
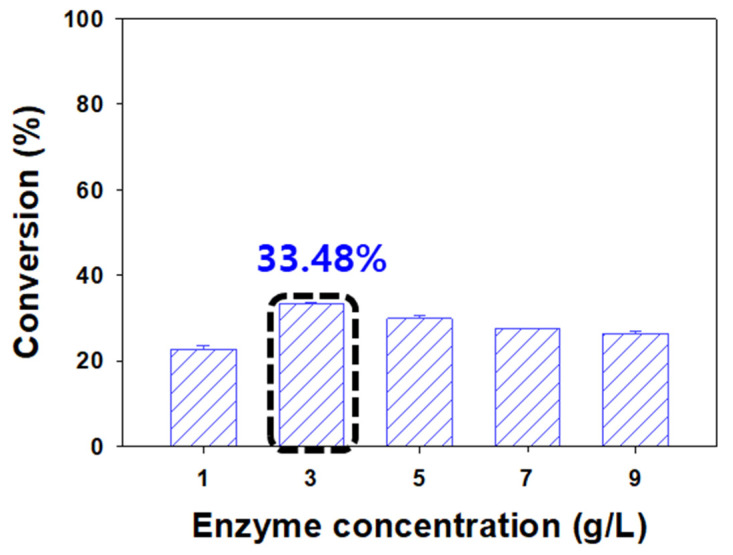
Effect of enzyme concentration. (Lipozyme TL IM, vinyl acetate as the acyl donor, 1:1 molar ratio of naringin to acyl donor, reaction temperature of 40 °C, *tert*-amyl alcohol as the solvent, and reaction time of 48 h).

As the amount of enzyme is an important economic factor, it is vital to obtain maximum efficiency using a small amount of enzyme. Therefore, to assess the production of naringin acetate according to the amount of enzyme, the concentration of Lipozyme TL IM was set to 1, 3, 5, 7, and 9 g/L. In our basal experiment, a control experiment without an enzyme was performed under the same reaction conditions. As a result, the conversion was less than 1%, and it was confirmed that the reaction did not proceed without the enzyme. Conversion in the presence of 1 g/L and 3 g/L increased to 22.77% and 33.48%, while that in the presence of 5 g/L decreased to 29.89%. The conversion reduced continuously thereafter, being 27.60% and 26.29% at 7 g/L and 9 g/L, respectively (Figure 4). Thus, 3 g/L was determined as the optimum enzyme concentration.

In an enzymatic reaction, a specific substrate and the active site of the enzyme are combined to form an enzyme-substrate complex, which initiates the reaction [14,20]. When the enzyme concentration is low, fewer active sites of enzymes are available to be used by the substrate; therefore, the conversion increases with the increased enzyme. According to the Michaelis–Menten equation, if the amount of enzyme relative to the substrate is small, the substrate becomes excessive, and the initial reaction rate increases after the enzyme concentration is increased [21,22,49]. Meanwhile, when the enzyme is present in excess, the excess active site is not exposed to the substrate [14,50,51], and the formation of the enzyme-substrate complex does not increase [48,51]. In addition, when the immobilized enzyme particles are present in excess, the dispersion power of the enzyme molecules decreases along with the number of effective collisions between molecules, thereby reducing the mass transfer rate [52,53,54]. A study on the external diffusion effect of an immobilized enzyme reaction revealed that the surface concentration of the substrate increases with the increase in the mass transfer rate, while the surface concentration of the product decreases in that order [55]. The concentration of the substrate or product is directly related to the rate of diffusion, which causes a change in the motion constant and impacts conversion. Hari Krishna et al. [50] reported the synthesis of isoamyl acetate using Lipozyme TL IM and found that the initial reaction rate decreased with an increase in enzyme concentration, with no difference observed in the conversion after 3 g/L. The authors had explained that when the enzyme is present in excess, excess active sites are not exposed to the substrate, and hence do not contribute to the reaction [14,20,21,22]. This is similar to our observation of a slight decrease in conversion at an enzyme concentration of 3 g/L.

### 3.4. Effects of Acyl Donors on the Conversion of Naringin Acetate

Naringin acetate is synthesized by the nucleophilic acyl substitution reaction between naringin and an acyl donor. To determine the effects of the acyl donors, experiments were conducted using acetic acid, acetic anhydride, methyl acetate, vinyl acetate, ethyl acetate, propyl acetate, and butyl acetate. Results showed a high conversion rate of 36.91% and 33.48% for acetic anhydride and vinyl acetate, respectively (Table 3). Therefore, we selected vinyl acetate and acetic anhydride as optimal acyl donors.

Acyl donor is a carbonyl compound with the electronegative atom as a leaving group. The stronger the leaving group, the more stable it is, by providing more electron pairs. However, the more stable it is, the lower its capacity to act as a leaving group. In general, the ability of the leaving group follows the order: acid chloride > anhydride > carboxylic acid = ester > amide. Therefore, anhydrides are more reactive than esters. In addition, as acetic anhydride has two carbonyl groups, it can perform a complex reaction [56,57]. First, it reacts with naringin to synthesize naringin acetate, with acetic acid produced as a by-product. The produced acetic acid, as a new acyl donor, reacts with the remaining naringin to further synthesize naringin acetate. Therefore, the highest conversion could be obtained with acetic anhydride as the acyl donor. Previous studies have reported the use of vinyl acetate as an acyl donor to produce various flavonoid acetates, including naringin acetate [10,58,59]. However, to date, the utilization of acetic anhydride as an acyl donor in flavonoid ester synthesis has not been reported, and this is the first study to do so.

Vinyl acetate also showed a high conversion rate, which can be explained by the tautomerization of by-products. When vinyl acetate is used as an acyl donor, vinyl alcohol is produced as a by-product, which is highly unstable in the form of an enol and is immediately converted to acetaldehyde by tautomerization. According to the results of Kim et al., by-products generated from the reaction could interfere with the reverse reaction, resulting in an irreversible flow [42,56].

By-products including residual acetaldehyde can be removed through purification steps. Zheng et al., reported that naringin esters were synthesized by the reaction of naringin with fatty acids, and that the high purity (>97%) of naringin esters was finally achieved through purification by two-step solvent extraction [27]. In this study, we focused on the effect of acyl donor on the synthesis of naringin acetate, and in our next study we will perform the optimization of the purification process to increase the purity of the target compound.

### 3.5. Effects of Molar Ratio and Reaction Temperature on the Conversion of Naringin Acetate

**Figure 5:**
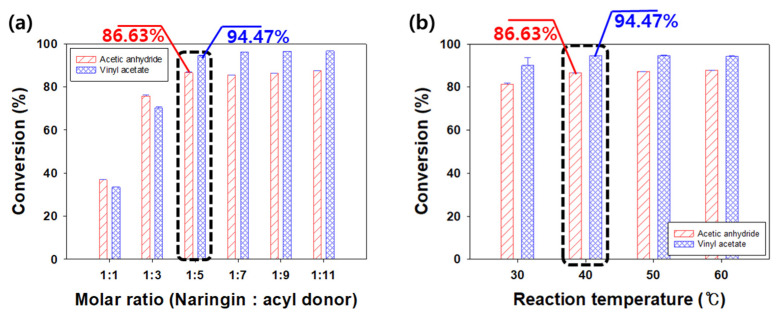
Effects of molar ratio between naringin and acyl donor (**a**), and reaction temperature on the conversion of naringin acetate (**b**).

Substrate concentration is an important factor in an enzymatic reaction [14,20,21,22,25]; therefore, we conducted experiments by altering the molar ratio of the reactants. We altered the molar ratio of naringin and the acyl donor (1:1, 1:3, 1:5, 1:7, 1:9, and 1:11) and determined its effect on the conversion of naringin acetate (Figure 5a). The enzymatic reaction was conducted at 40 °C using 3 g/L Lipozyme TL IM with *tert*-amyl alcohol for 48 h.

In the case of acetic anhydride, the conversion increased significantly to 36.91% and 75.72% at 1:1 and 1:3, respectively, and increased further to 86.63% at 1:5. Thereafter, conversions of 85.61%, 86.41%, and 87.19% were obtained at 1:7, 1:9, and 1:11, and were similar to that at 1:5.

In the case of vinyl acetate, the conversion increased to 33.48%, 70.29%, and 94.47% at 1:1, 1:3, and 1:5, respectively. Thereafter, a 96.07%, 96.55%, and 96.70% conversion was obtained at 1:7, 1:9, and 1:11, respectively. As seen for acetic anhydride, the results obtained with vinyl acetate did not change significantly at molar ratios below 1:5.

As transesterification using lipase is a reversible reaction, an excessive acyl donor concentration is required for the reaction equilibrium to shift to transesterification [25,56]. The mechanism of transesterification of the acyl donor and naringin through lipase is as follows. First, serine as a nucleophile at the active site of lipase attacks the acyl donor to form a tetrahedral intermediate. The latter attacks histidine, resulting in the formation of a by-product. Next, the alcohol of naringin, as a nucleophile, attacks the intermediate, creating a new tetrahedral intermediate. Thereafter, the product naringin acetate is produced by the movement of electrons, and the active site of lipase returns to its original state. According to the mechanism [20,21,22,60], the acyl donor reacts with the enzyme first and then naringin reacts to form naringin acetate. If there is an excess of acyl donor, it will react more with the active site of the enzyme to form an intermediate, which then reacts with naringin to increase the conversion of naringin acetate. However, if an excessive amount of naringin is present, it binds to the active site of lipase and competitively inhibits it, forming a dead-end complex [14,60,61]. Chebil et al. had conducted experiments by varying the molar ratio of vinyl acetate/quercetin to 5, 10, 20, and 40, and reported conversions of 55%, 88%, 96%, and 96%, respectively [59]. As the ratio of vinyl acetate increased, the conversion rate also increased, before finally saturating. Due to the excess vinyl acetate, the molar ratio of the substrate was considered to affect the thermodynamic shift of equilibrium.

When the molar ratio of naringin to acyl donors was 1:5 or lower (1:7, 1:9, and 1:11), according to the increasing concentration of the acyl donor, the conversion was greater with vinyl acetate than with acetic anhydride. When acetic anhydride reacts with naringin, acetic acid is produced as a by-product. Since acetic acid is a strong acid, it can act as an inhibitor of enzyme activity, causing dead-end inhibition, and can inactivate enzymes by acidifying the micro-aqueous layer of the enzyme [49]. In the presence of excess acetic anhydride, acetic acid production was increased as a by-product. Beyond the molar ratio of 1:5, acetic anhydride appeared to have a lower conversion than vinyl acetate owing to the formation of acetic acid.

In an enzymatic reaction, temperature is a vital factor, influencing the activity and denaturation of the enzyme as well as the substrate solubility. As the temperature rises, collisions between enzymes and substrate molecules increase, which increase the reaction rate. In addition, as the reaction temperature increases, the viscosity of the solution decreases, and the solubility of the substrate and product increases [20,21,22,62,63]. However, at very high temperatures, the non-covalent bonds that stabilize the three-dimensional structure of the protein may be weakened, and the activity of the enzyme may be lost [56,64].

Our experiments were conducted at 30, 40, 50, and 60 °C using 3 g/L Lipozyme TL IM with *tert*-amyl alcohol under the determined molar ratio (1:5) for 48 h. We aimed to determine the effect of the reaction’s temperature on the production of naringin acetate; the results with acetic anhydride showed conversions of 81.25% at 30 °C and 86.63%, 87.13%, and 87.68% at 40, 50, and 60 °C, respectively, which was a slight difference. The conversions rate obtained with vinyl acetate were 90.03%, 94.47%, 94.65%, and 94.40%, respectively, similar to that with acetic anhydride (Figure 5b).

Based on the above results, the change in conversion was large when the temperature was increased from 30 °C to 40 °C, with only minimal changes observed thereafter. Therefore, the solubility of the substrate had the greatest influence on this reaction. Qian et al. investigated the effect of temperature in the range of 40–60 °C on the synthesis of isoorientin ester using free *Candida rugosa* lipase [65]. The conversion was observed to increase rapidly at 50 °C, with the maximum conversion of 62.6% obtained at 60 °C, and decrease sharply thereafter to 31% at 65 °C. They reported the solubility of isoorientin in the reaction system to increase with increased temperature. Khor et al. investigated the effect of the reaction’s temperature on biodiesel synthesis using Lipozyme TL IM [64]. Similar to our current findings, the reaction rate was reported to be increased when the temperature increased from 30 to 40 °C, and the optimum reaction temperature was 40 °C [14,64].

### 3.6. Effect of Solvent on the Conversion of Naringin Acetate

The use of organic solvents in the reaction medium shifts the thermodynamic equilibrium such that it favors ester synthesis over hydrolysis. In addition, most organic solvents have a lower boiling point than aqueous media, and hence are readily removed, being advantageous in the separation and purification processes [15,20,21,22]. This study used an organic solvent as the reaction medium to induce ester synthesis. Organic solvents affect the activity, stability, and denaturation of the enzyme. They also affect the solubility, regioselectivity, and stereoselectivity of the substrate. Therefore, the conversion of naringin acetate depending on the type of organic solvent was next evaluated. Lipase is known to exhibit a high activity and stability in hydrophobic organic solvents. However, hydrophobic solvents are not suitable for the synthesis of hydrophilic hydrocarbons or sugar esters [66]. Therefore, relatively hydrophilic organic solvents, such as acetonitrile, 1,4-dioxane, acetone, THF, *tert*-butyl alcohol, *tert*-amyl alcohol, and 1,2-dichloroethane were screened in this study.

When acetic anhydride was used as an acyl donor, the highest conversion was 98.51% in acetonitrile. Acetone and THF showed similar conversions, of 98.37% and 98.20%, whereas 1,2-dichloroethane showed the lowest conversion, of 18.03%.

When vinyl acetate was used as the acyl donor, the highest conversion was obtained with acetonitrile (98.49%), followed by *tert*-amyl alcohol and *tert*-butyl alcohol (94.47% and 91.16%, respectively). Similar to acetic anhydride, 1,2-dichloroethane showed the lowest conversion, of 37.32% (Table 4).

Log *p* is the logarithm value of the partition coefficient between water and octanol, representing the degree of hydrophobicity. The larger the log *p* value, the higher the hydrophobicity. The log *p* value of naringin was −0.44, whereas those of acetic anhydride and vinyl acetate were −0.27 and 0.73, respectively. The various reaction solvents examined, along with their log *p* values, were: acetonitrile (−0.33), 1,4-dioxane (−0.27), acetone (−0.16), THF (0.49), *tert*-butyl alcohol (0.58), *tert*-amyl alcohol (1.09), and 1,2-dichloroethane (1.48). A log *p* value < 2 indicates a hydrophilic organic solvent, whereas a value ≥ 4 indicates a hydrophobic organic solvent [20,21,22,67]. The dielectric constant represents solvent polarizability. In general, a high dielectric constant refers to polar solvents, while a low dielectric constant refers to non-polar solvents. Based on the increasing dielectric constant values, the reaction solvents could be arranged in the following order: 1,4-dioxane (2.25), *tert*-amyl alcohol (5.78), THF (7.5), 1,2-dichloroethane (10.4), *tert*-butyl alcohol (10.9), acetone (20.7), and acetonitrile (37.5).

In lipase-based reactions, such as biodiesel synthesis, hydrophobic solvents such as hexane and toluene are used. However, naringin is a polar, hydrophilic material with a very low log *p* value and does not dissolve in a hydrophobic solvent. Previous studies using Novozym 435 had shown it to be stable in hydrophilic solvents, such as acetonitrile or acetone [68]. From the results of the current study, Lipozyme TL IM was also confirmed to be stable in a hydrophilic organic solvent. Milisavljevic et al. had reported a 54.65%, 28.98%, and 15.72% conversion using acetonitrile, acetone, and *tert*-butyl alcohol, respectively, in the synthesis of phloridzil oleate [68]. The use of isooctane and dodecane showed very low conversions due to their low solubility in hydrophobic media. The current study showed the highest conversion in acetonitrile and a remarkably low conversion in relatively hydrophobic 1,2-dichloroethane. The solubility of naringin was low in 1,2-dichloroethane, and the reaction did not occur in more hydrophobic solvents (hexane, toluene, etc.), as it did not dissolve in these solvents. Hazarika et al. had investigated the influence of the hydrophobicity, polarization rate, and water solubility of the solvent on the initial reaction rate of the lipase reaction [69] and found less hydrophobic solvents, i.e., those with a low log *p* value, to have a high initial reaction rate, as the substrate was more partitioned between the active site of lipase and the solvent. In addition, the enzyme remained tighter at a low dielectric constant than at a high dielectric constant. For the initial reaction rate, the degree of hydrophobicity was judged as the most important factor, and the polar solvent was found to be a good reaction solvent in the transesterification reaction. The optimal solvent selected in this study was acetonitrile, with a very low log *p* value of −0.33 and a high dielectric constant of 37.5, making it a polar, hydrophilic solvent.

**Figure 6:**
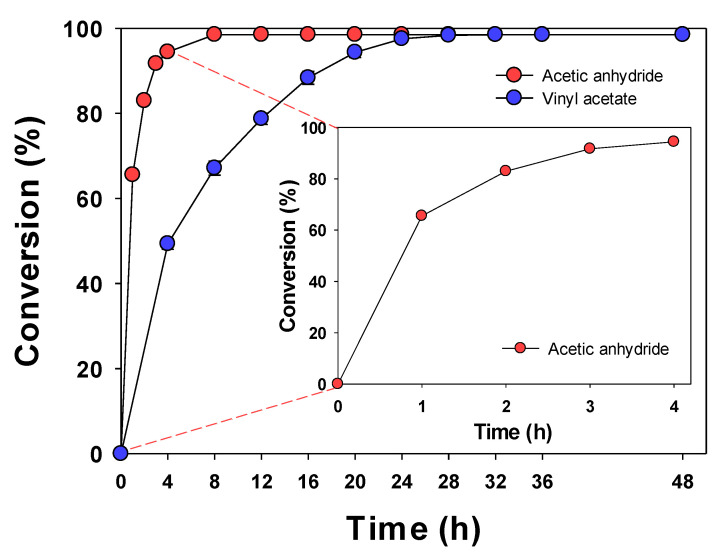
Conversion of naringin acetate as a function of reaction time (3 g/L of Lipozyme TL IM, acetic anhydride or vinyl acetate as the acyl donor, 1:5 molar ratio of naringin to acyl donor, reaction temperature of 40 °C, acetonitrile as the solvent, and reaction time of 48 h).

The conversion with respect to reaction time was investigated under the optimal conditions obtained through the OFAT method described above (Figure 6). The reaction was completed within 8 h when acetic anhydride was used, and within 24 h when vinyl acetate was used; each conversion was 98.51% and 97.54%, respectively. When acetic anhydride was used, the reaction appeared to be faster, since two reactions could occur. In a previous study by Romero et al., the synthesis of isoamyl acetate was confirmed to be faster with acetic anhydride than with any other acyl donor [49]. Previous studies on the synthesis of flavonoid esters required a long reaction time of more than 96 h [6,10,26,27,28,37,68,70,71]. Comparing the previous studies with the current one, the reaction time was found to be indeed shortened.

Table 5 summarizes the reaction conditions used in various studies to synthesize naringin esters through the enzymatic reaction of naringin and various acyl donors. Large amounts of enzyme and acyl donor were used in many studies, and a low conversion was obtained after a long reaction time. In contrast, this study used a small amount of enzyme (Lipozyme TL IM) and acyl donor (acetic anhydride or vinyl acetate) for the synthesis of naringin ester, and a high conversion was obtained in a short reaction time.

Based on the selection of major variables and the determination of optimal conditions, a significant conversion of approximately 98% of naringin acetate was achieved, starting from a conversion of less than 30%. In particular, when acetic anhydride is used as the acyl donor, the reaction time can be significantly shortened to within 8 h, and it can be confirmed that the conversion is at the highest level compared to previous reports. Naringin esters with improved bioavailability through acylation have a high potential in various fields such as food, cosmetics, and pharmaceuticals, but there are barriers to industrial applications due to a low conversion and a long reaction time. The improved enzymatic conversion and reaction time obtained through the conditions used in this study will contribute to the commercialization of naringin acetate. In the future, the evaluation of enzyme reuse based on stability will be performed under the determined reaction conditions, and it is expected that a completed enzymatic conversion process will be derived.

## 4. Conclusions

This study aimed to improve the enzymatic conversion of naringin acetate by screening important variables and determining the optimal reaction conditions. Under optimal conditions, the maximum conversions from acetic anhydride and vinyl acetate were found to be 98.5% (8 h) and 97.5% (24 h), respectively. The use of acetic anhydride as an acyl donor and Lipozyme TL IM as a biocatalyst was first attempted in the study of naringin acetate. Compared with the previous study, a high conversion rate could be achieved in a relatively short reaction time through the optimization of important variables. These results could potentially contribute to the economical and efficient synthesis of flavonoid esters using enzymes.

## Figures and Tables

**Table 1:** Summary of naringin ester conversion by enzymatic reaction.

Reactant		Reaction Type	Reaction Time	Conversion	Ref.
Naringin	Oleic acid	Esterification	48 h	93.10%	[14]
Naringin	Coconut oil	Esterification	90 h	75.43%	[25]
	Linseed oil			76.70%	
	Sunflower oil			85.08%	
Naringin	Ricinoleic acid	Esterification	120 h	33%	[26]
Naringin	Oleic acid	Esterification	96 h	78.4%	[27]
	Linoleic acid			77.6%	
	Linolenic acid			86.6%	
Naringin	Oleic acid	Esterification	96 h	80–90%	[28]
	Lauric acid				
	Linolenic acid				
Naringin	Vinyl acetate	Trans-esterification	96 h	41.03%	[10]
	Vinyl octanoate			91.40%	
Naringin	Vinyl butyrate	Trans-esterification	144 h	90%	[12]
Naringin	Vinyl laurate	Trans-esterification	8 h	50%	[29]

**Table 2:** Information on immobilized lipase and the corresponding conversion rate of naringin acetate.

Immobilized Lipase (Regioselectivity)	Source	Support	Conversion (%)
Novozym 435 (Nonspecific)	*Candida antarctica* lipase B	Acrylic resin	17.06 ± 0.72
Lipozyme TL IM (1,3-specific)	*Thermomyces lanuginosus*	Silica resin	29.89 ± 0.07
Lipozyme RM IM (1,3-specific)	*Rhizomucor miehei*	Anion-exchange resin	19.91 ± 0.20

(5 g/L of the enzyme, vinyl acetate as the acyl donor, 1:1 molar ratio of naringin to acyl donor, reaction temperature of 40 °C, *tert*-amyl alcohol as the solvent, and reaction time of 48 h).

**Table 3:** Information on acyl donors and the corresponding conversion rate of naringin acetate.

Acyl Donor	Molecular Formula	Structure	Molecular Weight (g/mol)	Conversion (%)
Acetic acid	C_2_H_4_O_2_	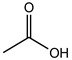	60.05	15.60 ± 0.24
Acetic anhydride	C_4_H_6_O_3_	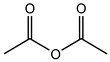	102.09	36.91 ± 0.35
Methyl acetate	C_3_H_6_O_2_	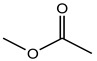	74.08	20.67 ± 0.90
Vinyl acetate	C_4_H_6_O_2_	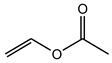	86.09	33.48 ± 0.28
Ethyl acetate	C_4_H_8_O_2_	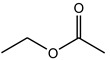	88.11	17.07 ± 1.04
Propyl acetate	C_5_H_10_O_2_	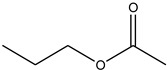	102.13	17.19 ± 0.60
Butyl acetate	C_6_H_12_O_2_	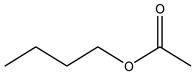	116.16	18.91 ± 0.62

(3 g/L of Lipozyme TL IM, 1:1 molar ratio of naringin to acyl donor, reaction temperature of 40 °C, *tert*-amyl alcohol as the solvent, and reaction time of 48 h).

**Table 4:** Information on organic solvents and the corresponding conversion of naringin acetate.

Organic Solvent	Log *p*	Dielectric Constant	Conversion (%) ^a^	Conversion (%) ^b^
Acetonitrile	−0.33	37.5	98.51 ± 0.01	98.49 ± 0.01
1,4-Dioxane	−0.27	2.25	89.96 ± 0.19	78.36 ± 1.52
Acetone	−0.16	20.7	98.37 ± 0.03	75.67 ± 0.04
THF	0.49	7.5	98.20 ± 0.02	88.30 ± 3.99
*tert*-Butyl alcohol	0.58	10.9	81.11 ± 0.46	91.16 ± 0.23
*tert*-Amyl alcohol	1.09	5.78	86.63 ± 0.27	94.47 ± 0.02
1,2-Dichloroethane	1.48	10.4	16.01 ± 2.85	37.32 ± 3.21

(a: 3 g/L of Lipozyme TL IM, acetic anhydride as the acyl donor, 1:5 molar ratio of naringin to acetic anhydride, reaction temperature of 40 °C, and reaction time of 48 h; b: 3 g/L of Lipozyme TL IM, vinyl acetate as the acyl donor, 1:5 molar ratio of naringin to vinyl acetate, reaction temperature of 40 °C, and reaction time of 48 h).

**Table 5:** Summary of reaction conditions for the synthesis of naringin esters by lipase.

Acyl Donor	Enzyme	Enzyme Conc., Molar Ratio	Solvent	Reaction	Conversion	Ref.
		**(Naringin: Acyl Donor)**				
Vinyl acetate	Whole-cell catalyst	Whole-cell 50 mg/mL, 1:50	Organic solvent	50 °C, 96 h	41.03%,	[10]
Vinyl octanoate	*Aspergillus oryzae*				91.40%	
Vinyl butyrate	Novozym 435	Enzyme 80 g/L, 1:10	Acetone	60 °C, 144 h	90%	[12]
Oleic acid	Lipozyme TL IM	Enzyme 10 g/L, 1:20	Acetonitrile	40 °C, 48 h	93.10%	[14]
				40 °C, 24 h	92.17%	
Coconut oil	Novozym 435	Enzyme 5 g/L, 1:6	Acetonitrile	65 °C, 90 h	75.43%,	[25]
Linseed oil					76.70%,	
Sunflower oil					85.08%	
Ricinoleic acid	Novozym 435	Enzyme 20 g/L, 1:3	Acetone	50 °C, 120 h	33%	[26]
Oleic acid	Novozym 435	Enzyme 15 g/L, 1:4	Acetone: *tert*-amyl alcohol (2:1)	50 °C, 96 h	78.4%,	[27]
Linoleic acid					77.6%,	
Linolenic acid					86.6%	
Lauric acid	Novozym 435	Enzyme 12 g/L, 1:5	Acetone	45 °C, 96 h	80–90%	[28]
Oleic acid						
Linolenic acid						
Vinyl laurate	Novozym 435	Enzyme 17 g/L, 1:10	Acetonitrile	50 °C, 8 h	50%	[29]
Acetic anhydride	Lipozyme TL IM	Enzyme 3 g/L, 1:5	Acetonitrile	40 °C, 8 h	98.51%	This study
Vinyl acetate	Lipozyme TL IM	Enzyme 3 g/L, 1:5	Acetonitrile	40 °C, 24 h	97.54%	This study

## Data Availability

The data are contained within the article.
